# Georgian Grapevine Cultivars: Ancient Biodiversity for Future Viticulture

**DOI:** 10.3389/fpls.2021.630122

**Published:** 2021-02-05

**Authors:** Maryam Sargolzaei, Laura Rustioni, Gabriele Cola, Valentina Ricciardi, Piero A. Bianco, David Maghradze, Osvaldo Failla, Fabio Quaglino, Silvia L. Toffolatti, Gabriella De Lorenzis

**Affiliations:** ^1^Dipartimento di Scienze Agrarie e Ambientali, Università degli Studi di Milano, Milan, Italy; ^2^Dipartimento di Scienze e Tecnologie Biologiche ed Ambientali, Università del Salento – Centro Ecotekne, Lecce, Italy; ^3^Faculty of Viticulture and Winemaking, Caucasus International University, Tbilisi, Georgia; ^4^National Wine Agency of Georgia, Tbilisi, Georgia

**Keywords:** *Vitis vinifera* L., genetic diversity, phenotypical characterization, resistance to diseases, climate change

## Abstract

Grapevine (*Vitis vinifera*) is one of the most widely cultivated plant species of agricultural interest, and is extensively appreciated for its fruits and the wines made from its fruits. Considering the high socio-economic impact of the wine sector all over the world, in recent years, there has been an increase in work aiming to investigate the biodiversity of grapevine germplasm available for breeding programs. Various studies have shed light on the genetic diversity characterizing the germplasm from the cradle of *V. vinifera* domestication in Georgia (South Caucasus). Georgian germplasm is placed in a distinct cluster from the European one and possesses a rich diversity for many different traits, including eno-carpological and phenological traits; resistance to pathogens, such as oomycetes and phytoplasmas; resistance to abiotic stresses, such as sunburn. The aim of this review is to assess the potential of Georgian cultivars as a source of useful traits for breeding programs. The unique genetic and phenotypic aspects of Georgian germplasm were unraveled, to better understand the diversity and quality of the genetic resources available to viticulturists, as valuable resources for the coming climate change scenario.

## Grapevine: A High Socio-Economic Impact Crop Strongly Threatened by Climate Change

The genus *Vitis* is present in 10 distribution areas, all in the northern hemisphere: five in North America, where 29 species have been described; four in Asia, with at least 11 species; and only one, *Vitis vinifera*, in a wide range that includes the Mediterranean, sub-Mediterranean, and Caucasian floristic regions with a spread toward the Pontic, Caspian, and Central Asiatic areas (Mullins et al., 1992). *V. vinifera* is one of the most widely cultivated plant species of agricultural interest and the only species extensively used in the global wine industry, covering approximately 7.4 Mha in 2018, and producing more than 77.8 mt of grapes (wine, table and dried grapes) and a world wine trade worth around EUR 32 billion^1^. Regions of its cultivation are located roughly between the 35th and 55th northern parallels and between the 25th and 35th southern parallels, in areas with average annual temperatures between 10 and 20°C. These environments are characterized by the alternation of a favorable growing season and an unfavorable cold one. However, the cold winters are not too intense (minimum temperatures range between −10 and 15°C) and the favorable season (average temperature higher than 10°C) is long enough (>200 days) for grapes to ripen (Gladstones, 1992).

Viticulture depends on environmental resources (i.e., climate and soil conditions) in terms of yields and quality (van Leeuwen and Darriet, 2016). The current climatic phase, characterized by the increase of average global temperature, has led to changes in the environmental conditions of agricultural areas that need to be tackled with suitable tools, in a context of adaptation and mitigation. Due to the socio-economic impact of the wine sector in Europe and around the world, over recent years, there has been an increase in work aiming to study the impact of climate change on viticulture (Hannah et al., 2013; Morales-Castilla et al., 2020).

**FIGURE 1:**
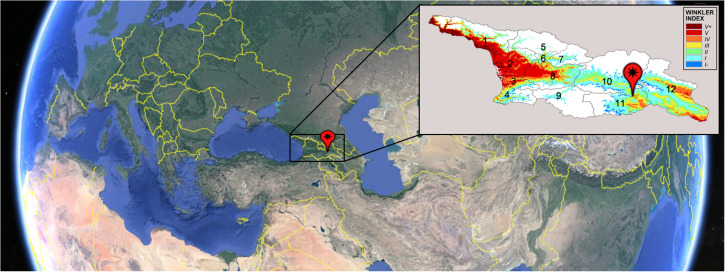
Map of Georgia and location of 12 Georgian wine-growing regions: 1 – Abkhazeti; 2 – Samegrelo; 3 – Guria; 4 – Adjara; 5 – Svaneti; 6 – Lechkhumi; 7 – Racha; 8 – Imereti; 9 – South Kartli; 10 – Inner Kartli; 11 – Lower Kartli; 12 – Kakheti. Image is obtained by Google Earth. Pin indicates Tbilisi position. Map on the right reports the Winkler classification based on yearly average Winkler index calculated for the period 1994–2013 in Georgia (Caucasus). The analysis is limited to the areas below 1250 m above sea level. Description of Winkler indices: (I–) GDD (Growth Degree Days) < 850, viticultural climate is very cool, vinicultural aptitude is very early ripening grapes for fresh and fruity wines or sparkling wine bases. (I) GDD 850–1400, viticultural climate is cool, vinicultural aptitude is early ripening grapes for fresh and fruity wines or sparkling wine bases. (II) GDD 1400–1650, viticultural climate is temperate cool, vinicultural aptitude is early ripening grapes for wines to be aged. Medium ripening grapes for white or red wines ready to drink. (III) GDD 1650–1950, viticultural climate is temperate, vinicultural aptitude is medium ripening grapes for white or red wines ready to be aged. (IV) GDD 1950–2200, viticultural climate is temperate warm, vinicultural aptitude is late ripening grapes for white or red wines ready to be aged. (V) GDD 2200–2700, viticultural climate is hot, vinicultural aptitude is late ripening grapes for bodied red wines to be aged. (V+) GDD > 2700, viticultural climate is very hot; vinicultural aptitude is very late ripening grapes for bodied red wines to be aged.

Santos et al. (2020) proposed a list of measures to be adopted in viticulture to face with the climate change. The list divided the measures in two categories: the short-term adaptation strategies and the long-term adaptation strategies. The short-term strategies include crop cultural practices and techniques to delay ripening time, plant protection against extreme heat, irrigation, pest and disease control and soil management. Among the long-term strategies, there are: change in training systems, varietal/clonal and rootstock selection and vineyard relocation. Breeding programs for new varieties which will be better able to perform in the environmental conditions expected in the future could be one of the most promising solutions, although this strategy is included in the long-term ones. An appropriate cultivar selection reduces the inputs required for plant management, increasing the sustainability of production. Great sources of biodiversity in the *V. vinifera* species have been recently found in its domestication cradle, located in Georgia (South Caucasus) (Imazio et al., 2013) (Figure 1).

Georgia counts 48,000 hectares of vineyards and a production of wine and table grapes of 159,000 and 8,000 tons, respectively (see text footnote 1). In 2015, about 100 M liters of wine were produced, 80% of them obtained by white and 20% from red berry grapes. More than 90% of the 2015 production was supported by Kakheti region, producing mainly white and red wines from Rkatsiteli and Saperavi grapes, in the ratio 7:3.

The aim of this review is to assess the potential of Georgian cultivars as sources of useful traits for new breeding programs, aiming to face the future challenges that await viticulture worldwide. To do this, we reviewed the particular genetic and phenotypic aspects (such as berry traits and resistance to pathogens) of Georgian germplasm, in the hope of better understanding the diversity and quality of the genetic resources available to viticulturists, coming directly from the origin of domestication.

## South Caucasus, the First Grapevine Domestication Center

*Vitis vinifera* is indigenous to Eurasia and it is suggested that the ancestors of the first *Vitis* genus appeared about 65 million years ago (Olmo et al., 1995). Nowadays, *V. vinifera* species includes both cultivated (*V. vinifera* subsp. *sativa*) and wild (*V. vinifera* subsp. *silvestris*) subspecies, the latter considered the progenitor of subspecies *sativa* (This et al., 2006). Its domestication process seems to be strongly linked to the alcoholic and gustative superiority of its fermented juice (the wine) in comparison to that of other fleshy fruits (fruit wines), although it is not well known which process predated the other (Terral et al., 2010). The main changes driving grapevine domestication were identified in the flower morphology (appearance of hermaphrodite flowers), larger berry size, higher berry sugar content, a wide range of berry color and aromatic content, characters which ensure yield, quality and a greater sugar content for a better fermentation (This et al., 2006). The major questions about grapevine domestication process are related to the number of events occurred, single event *versus* multiple events, and the geographical location where these events took place. For a vine domestication center to be born, different conditions need to occur. Among these, there is a strong awareness in practicing and developing viticulture by entire peasant villages. To bring out such a situation, many factors have to converge: territories with a (relatively) high population density, with stable settlements and in positions at crossroads of trade flows and cultural trends (Forni, 2012). It would be reasonable to expect that such situation could have occurred in several areas, differing in chronology and level of development. The most accredited hypothesis suggests that *V. vinifera* was domesticated from its wild form in the South Caucasus, between the Caspian and Black Seas, around 6,000–5,800 BC, and then spread throughout Europe and Mediterranean areas thanks to the spread of civilizations (McGovern et al., 2017). Recently, Zhou et al. (2019), proposing a four-state domestication process for grapevine, date the beginning of this process around 20,000 years ago, when South Caucasian human populations started to manage and harvest the local wild populations (Stage 1). In the same region around 8,000 years ago, the humans started with the conscious or unconscious selection of desirable phenotypical traits (Stage 2), although this transition is not well documented yet. Another force driving the transition from Stage 1 to Stage 2 is the bottleneck. Nevertheless, genetic evidences showed that grapevine did not experience a severe bottleneck (Myles et al., 2011; Zhou et al., 2017), making the conscious or unconscious selection as a unique force shaping the genetic diversity of grapevine during the domestication process. Stage 3 consists on spreading of newborn crop in new locations and the consequence of local domestication or introgression events. Reviewing the most comprehensive studies on grapevine genetic population, it turned out that an East-to-West grapevine gene flow after the first domestication process occurred, with some evidence of putative secondary domestication centers along the main migration routes due to genetic relationships between wild and cultivated accessions, especially in the Mediterranean Basin and Central Asia (Grassi et al., 2003; Arroyo-García et al., 2006; Myles et al., 2011; Zhou et al., 2017; Riaz et al., 2018). The coexistence of wild populations together with domesticated ones is often and the bidirectional gene flow (wild-to-cultivated and cultivated-to-wild) has been well documented (De Andrés et al., 2012; Ekhvaia et al., 2014; Riaz et al., 2018; D’Onofrio, 2020; Maraš et al., 2020), supporting the occurrence of secondary domestication events from local wild populations or introgression events. These events, the geographical origin and human usage were found to strongly shape the genetic structure of grapevine germplasm (Bacilieri et al., 2013). Another aspect, although less investigated, is the role of wild *Vitis* species in the *sativa* domestication process. It seems that wild *Vitis* species have contribute to the current structure of grapevine germplasm (Zhou et al., 2019). The last stage proposed by Zhou et al. (2017) (Stage 4) takes into account the modern breeding programs, a relative recent event occurred over the last few hundred years and led to the birth of so-called anthropic crossings, with the aim of satisfying specific requirements.

## Georgian Territory, Climate, and Grapevine Production

Georgia is a large basin of the mid latitudes, bordered by the Greater Caucasus in the North and the Lesser Caucasus in the South, and opening toward the Black Sea in the West and toward the Caspian depression in the East (Figure 1). Those geographical features strongly characterize the climate of its 12 wine-growing regions that, following the Köppen – Geiger classification (Köppen and Geiger, 1936), are characterized by profoundly different climatic conditions, ranging from hot summer continental climates to warm summer continental or hemiboreal climates, that translate into different classes of the Winkler index (Figure 1).

In relation to the climatic conditions of each wine-growing region, the Georgian varietal assortment is strongly differentiated as well, being adapted to a very wide range of cold and summer stresses (Table 1). Worldwide wine-growing regions experiencing the same climatic conditions of Georgia may provide benefit by this so differentiated varietal spectrum.

**TABLE 1:** Georgian grape ranges in comparisons to the main abiotic stress.

Region	Risk of				Recommended varieties	
	Summer light-thermal stress^1^	Summer water stress^2^	Winter frost^3^	Spring frost^4^	Colored varieties^5^	White varieties^5^
Abkhazeti	Very high	Very low – low	Very low	Very low – high	Amlakhu N, Kachichi N, Absuaj N, Lakoaj N, Ojaleshi N, Chkhaveri N, Amlakhu N	Avasikhva B, Aghbizh B, Akabuli B, Khapshira B, Khunaliji B, Tsolikouri B, Krakhuna B
Samegrelo	Very high	Very low – low	Low – very low	Low – high	Ojaleshi N, Chvitiluri N	Chechipeshi B
Guria	Medium	Very low	Low	Very low – high	Chkhaveri N, Jani N, Mtevandidi N, Skhilatubani N	Sakmiela B
Adjara	Very low – low	Very low – low	Low – very low	Very low – low	Mekrenchkhi N, Burdzghala N, Jineshi N, Satsuri N, Batomura N	Brola B, Khopaturi B, Klarjuli B, Kviristava B, Shavshura B
Svaneti	Very low (high)	Very low	Very low – high	Very low – high	Alexandrouli N, Mujuretuli N, Orbeluri Ojaleshi N, Usakhelouri N, Rachuli Dzelshavi N	Tsulukidzis Tetra B, Tsolikouri B
Lechkhumi	High – very high	Very low – low	Very low	Low		
Racha	Very low – low – (high)	Very low – low	Very low – very high	Very low – very high		
Imereti	High – very high	Very low	Low	Very low – very high	Aladasturi N, Dzelshavi N, Otskhanuri Sapere N, Argvetuli Sapere N, Rko N, Adanasuri N, Bzvanura N, Dondghlabi Shavi N, Vani [or Vanura?] N, Chkhaveri N	Goruli Mtsvane B, Krakhuna B, Tsolikouri B, Tsitska B, Kvishkhuri (sin. Goruli Mtsvane) B, Dondghlabi B, Bazaleturi B, Kundza Tetri B, Tklapa B
South Kartli	Very low – very high	Low – medium	Very low – very high	Very low – very high	Tavkveri N, Asuretuli Shavi N, Shavkapito N, Saperavi Budeshuriseburi N, Saperavi N, Dzelshavi N	Chinuri B, Goruli Mtsvane B, Rkatsiteli B, Budeshuri B, Jvari B, Adreuli B, Aragvispiruli B, Grdzelmtevana B, Melikuda B, Chrola Kartlis B, Kharistvala B
Inner Kartli	High – very high	Medium	Very low –high	Very low –high		
Lower Kartli	Low – high	Medium	Very low	Very low – low		
Kakheti	Very high	Medium – high	Very low	Low – very low	Saperavi N, Saperavi Budeshuriseburi N, Tavkveri N, Budeshuri Tsiteli N, Ikaltos Tsiteli N	Rkatsiteli B, Kisi B, Mtsvane Kakhuri B, Khikhvi B, Muskaturi Rkatsiteli B, Chinuri B, Mtsvivani Kakhuri B, Sapena B, Kumsi Tetri B

***^1^ Summer Stress** (1974–2013): The risk of summer stress is expressed as the percentage of the years of the reference period with at least 7 days with maximum temperature above the 35°C threshold. The classes are: very low (<3%), low (3–5%), medium (5–6.7), high (6.7–10%) and very high (>10%). **^2^Water Shortage** (1974–2013) Calculated by means of a daily water balance. Average yearly sum of the stress level of stress days. The classes are: very low (0 day/year), low (0–15 days/year), medium (15–30 days/year), high (30–60 days/year), and very high (>60 days/year). **^3^ Winter Frost** (1974–2013): The risk of winter frost is expressed as the percentage of the years of the reference period winter minimum temperature below the −15°C threshold. The classes are: very low (<3%), low (3–5%), medium (5–6.7), high (6.7–10%) and very high (>10%). **^4^ Spring Frost** (1974–2013): The risk of spring frost is expressed as the percentage of the years of the reference period with spring minimum temperature below the −2°C threshold. The classes are: very low (<3%), low (3–5%), medium (5–6.7), high (6.7–10%), and very high (>10%). **^5^** N, noir; B, Blanc.*

It is interesting to highlight that, in 1994 Georgia faced an abrupt rise in temperatures, similarly to what happened in Western Europe in the late 1980s (Reid et al., 2016), with 1987 as the most likely year of change (Mariani et al., 2012). This delay could be explained by the progressive dilution of the Atlantic circulation signal as it moves into the European continent (Cola et al., 2017). The increase of temperature determined an advance in grapevine phenology, which was more significant at the higher altitudes, where more favorable thermal conditions were established. On the other hand, at lower altitudes the phenological advance was partially depleted by the increase of super-optimal thermal conditions (increasing the occurrence of stress conditions during ripening). For instance, in the case of the widely diffused cultivar Rkatsiteli, the average advance of veraison was less than 6 days for the 250–500 m asl elevation belt and around 18 days for the 750–1000 m one (Cola et al., 2017).

**FIGURE 2:**
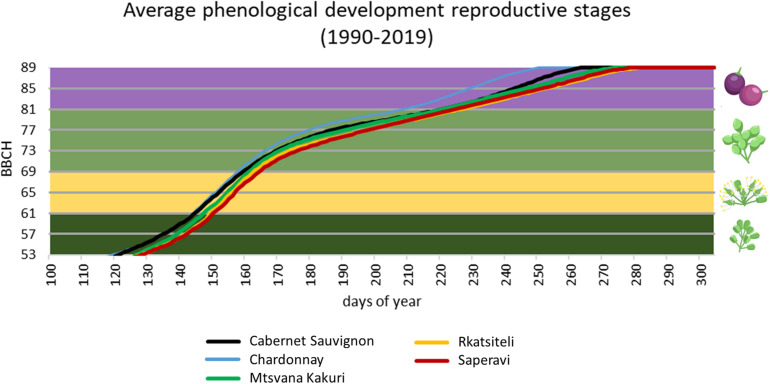
Phenological timing simulation for three relevant Georgian cultivars, compared with Chardonnay and Cabernet sauvignon, using meteorological data of Perugia (Italy) (years 1990–2019). Phenology is represented following the reference BBCH scale: (i) 53–59 development of flowers; (ii) 60–69 flowering; (iii) 70–79 development of fruits; (iv) 80–89 ripening.

In parallel, it is worth noticing the high variability in the plant phenology among Georgian cultivars, both in the sprouting date and in the ripening period. A delayed budburst period could represent an avoidance mechanism against spring frosts. Considering Georgian cultivars, bud swelling of ‘Partala’ vines was recorded at the end of March, and, thus, a higher susceptibility to spring frost is expected when compared to the other cultivars that sprouted in April (Maghradze et al., 2014). Global warming generally resulted in the increase of cases of temperature above the optimal range (24–26°C) during summer and in particular during grape ripening (Cola et al., 2020). A delay in the maturation process, obtained through the selection of late-ripening cultivars, could ensure thermal conditions during ripening more suitable for berry metabolism. Maghradze et al. (2012) studied the phenology of Georgian cultivars in northern Italy in comparison to Chardonnay and Cabernet Sauvignon grown in the same area, and they found a relatively late ripening with respect to the reference varieties: nevertheless, a very wide range of variability was observed. Similar results were found in other comparative evaluation carried out in Georgian ampelographic collections and are reported by Maghradze et al. (2014) and Rustioni et al. (2014). Some extreme cases are: early ripening cultivars – Kartuli Saadreo, Meskhuri Mtsvane, Buza, Budeshuri Tsiteli and Daisi; late ripening cultivars – Ojaleshi, Akomshtali, Kamuri, Shavi, Tavkara, Khushia Shavi, Satsuravi, Maghlari Tvrina, Mtevandidi, Argvetula, Dziganidzis Shavi, Adanasuri, Mamukas Vazi, Otskhanuri Sapere, Gorula, Saperavi Meskhuri, Ghrubela and Shavtita. The same results were obtained when comparing the phenological timing of Georgian varieties internationally grown. The phenological model developed by Mariani et al. (2013) for Cabernet Sauvignon and Chardonnay and adapted to the Georgian varieties Saperavi, Rkatsiteli, Mtsvane Kakhuri (Cola et al., 2017) was applied to a long time series of daily temperature (Perugia–Italy. 1990–2019). Figure 2 shows the late phenological timing of Georgian varieties (average values are shown).

## The Ampelographic Collections of Georgian Germplasm

To date, fifty grapevine varieties are recommended for cultivation in Georgia. Most of them (37) are wine grape cultivars, while the others (13) are generally used to produce grapes for fresh consumption. Predominantly, the Georgian vineyards are cultivated with autochthonous varieties: among the recommended wine grapes, 31 are local varieties and seven are international cultivars, while, considering the table grapes, nine of them have a local origin: four are traditional, autochthonous, Georgian cultivars, five are from local breeding outputs, and five are allochthonous varieties (Chkhartishvili and Maghradze, 2012). Beside the recommended autochthonous varieties, other cultivars enlarge the intraspecific biodiversity preserved in Georgia: Tsertsvadze (2012) described 48 grapevine native cultivars in the ‘Caucasus and Northern Black Sea Region Ampelography’ and further studies are in progress to continuously increase the number of recognized Georgian cultivars preserving this important source of biodiversity. Based on information available, more than 700 Georgian accessions can be counted (Supplementary Table 1), most of them are germplasm accessions and the rest are classified as major (20) and minor (8) cultivars. These accessions are available in nine Georgian collections (Table 2) and other collections hold by foreign Institutions, such as Italy (443 accessions), Ukraine (309 accessions), Russia (191 accessions), Moldova (122 accessions), Uzbekistan (32 accessions), France (20 accessions), and Slovakia (7 accessions). Although the number of accessions is high, only a limited number of them were genotyped and phenotyped (see sections “Georgian Germplasm as a Source of Genetic Variability” and “The Phenotypical Characterization of Georgian Germplasm Collections”). This limited number of information makes a not so easy determination of the exact number of autochthonous Georgian varieties. Further efforts are needed to better understand the genetic diversity of this valuable germplasm and to identify synonyms, homonyms and misidentifications.

**TABLE 2:** List of grapevine germplasm collections in Georgia.

Collection	Year of plantation	Total accessions	Old varieties	Foreign varieties	*Vitis vinifera* accessions	Rootstocks/non-*vinifera* species
Jighaura	2008	932	425	500	925	7
Mukhrani	2014	280	275	5	280	0
Skra 2	2008	330	330	0	330	0
Vachebi	2008	219	212	0	212	7
Telavi 2	2008	173	168	5	173	0
Shumi	2006	271	179	92	271	0
Kindzmarauli	2005	400	400	0	400	0
Telavi 1	1987	141	141	0	141	0
Skra 1	1972	75	38	37	75	0
Total		2821	2148	639	2807	14

### Georgian Germplasm as a Source of Genetic Variability

Historical information coupled with archeological and palaeobotanical findings pointed to Georgia as a cradle for grapevine domestication (Zohary and Hopf, 2000; McGovern, 2003; McGovern et al., 2017). Molecular analysis produced the same evidence. Genetic diversity of Georgian germplasm was investigated, by both nuclear SSR (simple sequence repeat) (Laucou et al., 2011; Imazio et al., 2013; Ekhvaia et al., 2014) and SNPs (single nucleotide polymorphisms) (De Lorenzis et al., 2015; Laucou et al., 2018) molecular markers, although a number of autochthonous cultivars, collected in local ampelographic collections, still remain to be studied (Supplementary Table 1). Thanks to two European research programs, GrapeGen06 (2007–2010) (Laucou et al., 2011), first, and then COST Action FA1003 (2011–2014) (Failla, 2015), a strong and still active network of scientific collaborations has been developed between European and Georgian researchers, to genetically characterize and preserve the Georgian genetic resources of vines.

**FIGURE 3:**
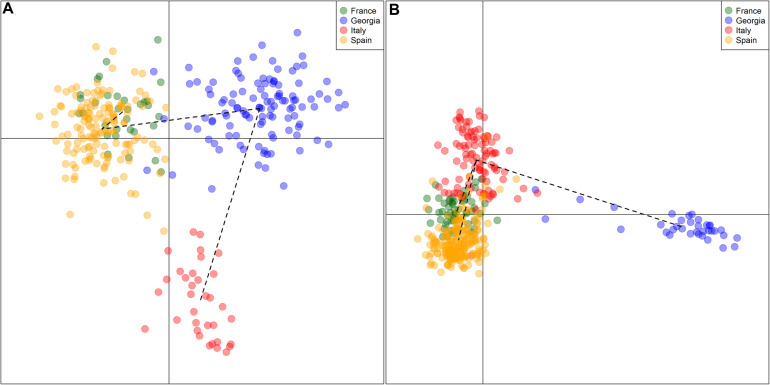
Two-dimension DAPC (discriminant analysis of principal component) scatter plot. Reworking of genetic profiles of grapevine cultivars coming from France, Georgia, Italy, and Spain, genotyped by 20 SSRs **(A)** and 18k SNPs **(B)**, using data reported in De Lorenzis et al. (2015), Laucou et al. (2018), Riaz et al. (2018), and De Lorenzis et al. (2019). DAPC was performed to identify genetic clusters using the package *adegenet* of R software. Black dotted lines represent a minimum-spanning tree.

All the outcomes about the genetic characterization of Georgian germplasm reported the uniqueness and originality of this germplasm when compared to the European and Central Asian germplasm (Myles et al., 2011; Bacilieri et al., 2013; Imazio et al., 2013; Riaz et al., 2018; De Lorenzis et al., 2019). The Georgian cultivars showed the distinctive features of a domestication center, such as high levels of genetic diversity and heterozygosity, the presence of alleles absent or poorly represented in other countries, and differentiation from the European varieties, clustering in a well-separated branch (as reported in the Figure 3, where SSR and SNP genetic profiles of varieties from France, Georgia, Italy and Spain were re-elaborated to perform a discriminant analysis of principal component, using data published in De Lorenzis et al., 2015, 2019, Laucou et al., 2018, and Riaz et al., 2018). A differentiation inside the germplasm, based on the geographical origin of cultivars, was identified as well: the varieties putatively originated in Kartli and Kakheti (Eastern regions) differ from the ones originating in Abkhazeti, Samegrelo, Guria, Adjara, Imereti, Racha, and Lechkhumi (Western regions). The origin of this subdivision lies in the geographical subdivision of Georgia into two major parts, due to the Likhi Mountains running in a North-to-South direction across Georgia (Imazio et al., 2013; De Lorenzis et al., 2015), confirming that, despite long-standing cultivation, the Georgian cultivars maintain their originality.

Genetic variation provides the foundation for any breeding programs, and natural genetic diversity represented historically the major source of variability for crop improvement and adaptation to changing environmental conditions. Given the uniqueness of Georgian germplasm, its strong link with the regions of origin coupled with the evidence of this country being the center of domestication makes this germplasm very attractive for investigation from the perspectives of phenology, grape phenotype and resistance to biotic and abiotic stresses, as sources of new variability for future breeding programs.

### The Phenotypical Characterization of Georgian Germplasm Collections

**FIGURE 4:**
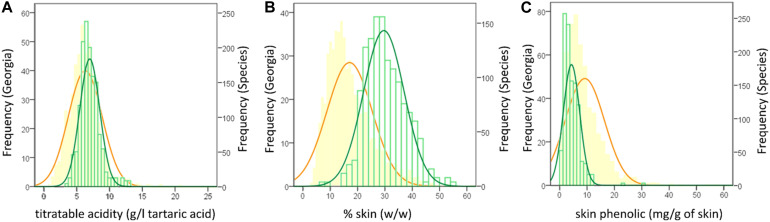
Frequency distribution of the entire *V. vinifera* species (orange) in comparison with the Georgian (green) records concerning the traits: titratable acidity **(A)**; % of skin **(B)** and skin phenolics **(C)** (reworking of the results published in Rustioni et al., 2014, 2019 and Abashidze et al., 2015).

The collaboration among European and Asian researchers makes feasible the comparisons among ampelographic collections using common protocols. Among them, phenotyping was considered in the framework of the COST Action FA1003 (Rustioni et al., 2014), allowing the description of numerous autochthonous cultivars (Abashidze et al., 2015; Cornea and Savin, 2015; Goryslavets et al., 2015; Maghradze et al., 2015; Ujmajuridze and Mamasakhlisashvili, 2015). This work, finally, produced a general overview of the *V. vinifera* variability concerning eno-carpological traits (Rustioni et al., 2019). Table 3 reports the distribution of the Georgian records with respect to the variability described for the *V. vinifera* species (data obtained by the reworking of the results published in Rustioni et al., 2014, 2019, and Abashidze et al., 2015). To emphasize some results, showing the differences among the two groups of data, Figure 4 shows the frequency distribution of the Georgian records in comparison with the data collected for the entire *V. vinifera* species, concerning some specific traits (titratable acidity, percentage of skin, skin phenolic content). Briefly, the main differences highlighted in Table 3 in terms of oenological applications are that Georgian grapes have, with respect to the *V. vinifera* species population, higher concentrations in both sugars and acids and thicker skins, ensuring acceptable amounts of phenolics despite the lower accumulation per unit of tissue. Details concerning the results of this comparison are discussed in Sections “Fruit Morphology and Technological Quality of Georgian Cultivars” and “Abiotic Stress Adaptations and Secondary Metabolisms.” It is worth noting that the phenotypic variability reported is due to the genotype, to the environmental growing conditions, and to their interactions. Thus, further studies will be necessary to discriminate these effects, highlighting the role of genotypes.

**TABLE 3:** Physical dimensions and chemical components distribution of Georgian grapevine germplasm with respect to the variability described for the entire *V. vinifera* species (reworking of the results published in Rustioni et al., 2014, 2019 and Abashidze et al., 2015).

Variable	Sample number		Average			Minimum		Maximum		Quartiles					
										25		50 – median		75	
	Georgia	Species	Georgia	Species	*Significance of the difference*^1^	Georgia	Species	Georgia	Species	Georgia	Species	Georgia	Species	Georgia	Species
Berry length (mm)	303	22383	14.16	15.02	0.000	9.3	5	19	37	12.9	13	14	15	15.5	17
Berry width (mm)	303	22385	13.03	14.18	0.000	9	6	17.5	29	11.6	12	13	14	14.3	16
Length/width	303	22383	1.10	1.06	0.000	0.93	0.50	1.39	3.60	1.04	1.00	1.08	1.00	1.14	1.10
Bunch weight (g)	261	5737	184.46	247.76	0.000	26	10	641	1362	109	143	167	220	229	319
Sugar content (Brix)	336	2162	21.51	20.8	0.073	12	10.0	28	35.0	20	19.0	21	21.0	24	23.0
Titratable acidity (g/l tartaric acid)	336	2161	7.01	6.3	0.005	3.5	0.8	13.2	22.7	6	4.7	6.8	6.0	7.8	7.4
Berry weight (g)	336	2404	2.20	2.4	0.037	0.8	0.6	4	10.1	1.7	1.6	2.2	2.2	2.6	2.8
% Skin (w/w)	334	2368	29.63	17.0	0.000	7	3.0	54	54.0	24	11.0	29	15.0	34	21.0
% Seed (w/w)	336	2355	4.24	4.0	0.047	2	0.0	10	17.0	3	3.0	4	4.0	5	5.0
Weight of 1 skin (g)	334	2369	0.64	0.4	0.000	0.1	0.1	1.3	1.9	0.5	0.2	0.6	0.3	0.8	0.5
Number of seeds/berry	336	2321	2.04	2.1	0.047	0.9	0.0	3.8	4.3	1.7	1.7	2	2.1	2.4	2.5
Weight of 1 seed (mg)	336	2293	44.17	41.0	0.000	20	10.0	90	160.0	40	30.0	40	40.0	50	50.0
Anthocyanins (mg/kg of grapes)	206	1141	756.55	710.1	0.699	50	50.0	3350	5350.0	350	200	600	550	950	1000
Anthocyanins (mg/berry)	204	1138	1.45	1.4	0.153	0.1	0.1	5	8.5	0.7	0.5	1.3	1.0	2	1.9
Anthocyanins (mg/g of skin)	204	1138	2.77	4.7	0.000	0.1	0.1	9.8	45.0	1.125	1.5	2.35	3.2	3.7	6.1
Skin phenolic (mg/kg of grapes)	336	1739	1182.23	1375.8	0.002	200	90.0	3780	6590.0	720	680	1030	1090	1590	1800
Skin phenolic (mg/berry)	336	1739	2.45	2.8	0.313	0.2	0.2	6.2	12.0	1.5	1.6	2.3	2.4	3.275	3.6
Skin phenolic (mg/g of skin)	334	1735	4.34	9.1	0.000	0.5	0.3	30.6	61.4	2.3	4.5	3.7	7.3	6	11.9
Seed phenolic (mg/kg of grapes)	335	1724	177.70	337.0	0.000	10	10.0	1050	4180.0	60	100	120	210	260	430
Seed phenolic (mg/berry)	316	1692	0.40	0.7	0.000	0.1	0.1	2.3	5.4	0.1	0.2	0.3	0.5	0.6	0.9
Seed phenolic (mg/g of seed)	324	1704	4.46	8.7	0.000	1	1.0	28	98.0	2	3.0	3	6.0	7	11.0
Seed phenolic (μg/seed)	335	1723	190.87	338.4	0.000	10	10.0	1350	5390	60	110	130	220	290	440
Skin phenolics (%)	335	1734	86.28	79.9	0.000	30	22.0	99	100.0	81	70.0	89	84.0	95	92.0
Seed phenolics (%)	335	1734	13.72	20.1	0.000	1	0.0	70	78.0	5	8.0	11	16.0	19	30.0
Total phenolics (mg/kg of grape)	335	1735	1360.60	1708.7	0.000	250	100.0	4200	9550.0	850	900	1200	1450	1750	2200
Total phenolics (mg/berry)	335	1737	2.83	3.4	0.004	0.3	0.3	6.9	12.3	1.9	2.1	2.7	3.0	3.7	4.3

*^1^Significance between Georgian and species values has been evaluated by ANOVA performed in SPSS v.25 software.*

#### Fruit Morphology and Technological Quality of Georgian Cultivars

Despite the wide variability for berry shapes within the species, Georgian grapes generally have round or slightly elongated small berries (Table 3) (OIV, descriptor 223). This is probably due to the ancient traditions that, during millennia of winemaking activities (Chkhartishvili and Maghradze, 2012; McGovern et al., 2017), favored the selection of wine grapes over table grapes.

Considering technological maturity, Georgian records generally show higher concentrations in both sugars and acids than foreign varieties (Table 3). In the perspective of climate change, the high sugar content could represent a problem, due to the risk of further increases related to the higher temperatures during the anticipated ripening periods (Keller, 2010; Mira de Orduña, 2010; van Leeuwen and Destrac-Irvine, 2017). The increased sugar concentrations expected in hot ripening conditions may cause growth inhibition or lysis in the yeasts responsible for wine fermentation (Mira de Orduña, 2010). Furthermore, high sugar stress could modify the yeast metabolisms, increasing the accumulation of by-products (such as glycerol and acetic acid) that, together with the increased alcoholic content, affects the wine perceptions of consumers and, thus, it could modify the expected characteristics of traditional wines (Mira de Orduña, 2010). However, the deviation of Georgian data with respect to the species variability concerning the sugar content is rather limited (significance of the difference = 0.073), neither covering the maximum records of the one obtained when analyzing wider grapevine genetic pools (Table 3) (Rustioni et al., 2019). Furthermore, the expected sugar content increase is usually ascribed to the earlier ripening anticipated in climate changed conditions (Palliotti et al., 2014; Martínez-Moreno et al., 2019), and it is important to remind the prevalence of late ripening cultivars among the Georgian grapevines (Maghradze et al., 2012). Finally, the sugar content seems to be well counterbalanced by the acidity (Table 3). The distribution of Georgian grapes concerning the titratable acidity, showed a right shift (Figure 4A), demonstrating the ability of these cultivars to keep a high acidic concentration despite the sweetness of the berries. This is a crucial point for viticulture adaptation to climate change, because high temperatures usually cause a decrease in acids, especially due to malic acid degradation (Keller, 2010; Mira de Orduña, 2010; van Leeuwen and Destrac-Irvine, 2017).

In the perspective of climate change adaptation, it is very interesting to note a particular feature of Georgian grapes concerning the proportions among skin, seeds and pulp, at the expense of the latter (Table 3). The Georgian records shown in Figure 4B, demonstrate an important shift toward thicker skins with respect to the general *V. vinifera* species. This is due to the lighter berries and heavier skins (Table 3). Considering the effect of climate change on the berries, a thicker skin could represent a more resistant barrier against the stressful environment. In fact, it has been shown that a possible adaptation to climate change could be related to berry skin thickening (van Leeuwen and Destrac-Irvine, 2017).

#### Abiotic Stress Adaptations and Secondary Metabolisms

Grape epicuticular waxes also have important protective roles against dehydration (Pangavhane et al., 1999; Di Matteo et al., 2000; Doymaz, 2006; Muganu et al., 2011) and pathogen infections (Marois et al., 1986; Rosenquist and Morrison, 1988; Percival et al., 1993). Furthermore, a study conducted on Georgian cultivars, suggested a possible eco-physiological role of epicuticular waxes in reducing heating stresses by an interaction with infrared radiation (Rustioni et al., 2012). However, a comparison among Georgian cultivars and grape varieties cultivated in other regions is not available and, thus, we should suppose that this mechanism is not exclusive for Georgian cultivars.

Excesses of photosynthetically active radiation (PAR) could causeproblems in grapes due to chlorophyll overexcitation (Rustioni et al., 2015; Rustioni, 2017). Rkatsiteli response to photo-oxidativesunburn was tested by Rustioni et al. (2015). It was considered among the “tolerant cultivars,” as it showed relatively low susceptibility to sunburn (recorded asbrowning appearance) at all the phenological periods studied. Inparticular, the correlation between chlorophyll contents and browningsymptoms had a high *R*^2^ (0.989), but the slopecoefficient (60.2) together with the average Browning Intensity Index(27.5) indicated a light symptom appearance in Rkatsiteli grapes. Inphoto-oxidative sunburn, browning symptoms appear due to the reactiveoxygen species (ROS) scavenging activity of phenolics through theiroxidation and consequent polymerization that produce brown pigments(Felicetti and Schrader, 2008; Rustioni et al., 2015). Often, plants face stresses through secondary metabolites, and the crucial role of phenolics against photodamage is well known (Close and McArthur, 2002; Graham et al., 2004; Rustioni, 2017). However, if the substrate for these oxidative polymerizations (phenolics) are in low concentrations, the sunburn browning symptoms could appear less intense: this is likely in the case of Rkatsiteli. Abashidze et al. (2015) reported 404.7 ± 58.3 mg/kg of grapes as average skin phenolics for this cultivar, which falls in the first 10th percentile of the *V. vinifera* variability concerning this trait (Rustioni et al., 2019) (Table 3). Considering total phenolic compounds, Georgian cultivars appeared to accumulate low amounts of these molecules in skins (Figure 4C and Table 3), but the difference is still exacerbated by seed phenolics (Table 3). In fact, the average percentage of phenolics arising from seeds is much lower in data coming from Georgia (13.7% in Georgian records in comparison with 20.1% of species characteristics). Of course, considering the eco-physiological role of phenolics, this trait could be considered as a downside of Georgian cultivars. However, in a production perspective, it could be an important advantage.

Climate changes often produce disequilibria in the berry ripening processes, increasing the quantity of phenolic compounds (Keller, 2010; van Leeuwen and Destrac-Irvine, 2017) that, often, do not reach an optimal ripening quality. Unripe phenolics could strongly compromise the wine quality, being involved in the perception of bitterness and astringency (Kontoudakis et al., 2011). Seed phenolics, due to their intrinsic characteristics, are often considered as an undesirable source of defects, so it is true that technologies have been developed to separate seeds to prevent phenolic extractions in wines (Canals et al., 2008) or to artificially ripen them under controlled conditions (Rustioni et al., 2018; VanderWeide et al., 2020). In this perspective, and considering that climate change is expected to make it harder to reach an equilibrated phenolic ripening in grapes, the lower phenolic concentration of Georgian cultivars (especially in the seeds) could be considered as a positive trait to deal with future difficult ripening conditions.

Another important class of phenolic molecules is represented byanthocyanin pigments. Among the 48 native Georgian grapevinevarieties described by Tsertsvadze (2012), 21 of them are white grapecultivars, while the other 27 have pigmented berries (22 black, 2 red, 1 gray, and 2 pink). Among the native Georgian grapevarieties described by Ketskhoveliet al. (1960), 245 of them are not pigmented (241 white and 4 yellow) grape cultivars, while the other 278 have pigmented berries (221 black, 27 red, 5 gray, and 25 pink). The reflectance spectra of 51 Georgian cultivars, together with other 69 accessions originated from other countries, were studied by Rustioni et al. (2013). Based on this first screening, some of these cultivars were selected to highlight dysfunctions in anthocyanin accumulation: Ubakluri, Ghrubela Kartlis, Rkatsiteli Vardisperi (and Marguli Sapere among the reference cultivars). Ubakluri shows a very light color, due to a very low pigment accumulation. Ghrubela Kartlis, due to the prevalence of peonidin-3-*O*-glucosides among the anthocyanins, has a gray appearance. Rkatsiteli Vardisperi, with the salmon pink color due to the high proportion of cyanidin-3-*O*-glucosides, is considered a berry color mutant resulting from a retro-transposon-induced mutation of the Rkatsiteli white-skinned cultivar (Rustioni et al., 2016; De Lorenzis et al., 2020). These color peculiarities could be interesting for future selections, especially considering the importance of appearance for table grape markets.

The environmental conditions (e.g., light and temperature) can affect the pigment accumulation in skins and the modulation of the anthocyanin biosynthetic pathway in berries could be considered as a grapevine eco-physiological adaptation mechanism (Keller, 2010; Rustioni et al., 2011; De Lorenzis et al., 2016). Considering anthocyanins (Table 3), Georgian data generally show slightly higher contents of pigments with respect to the *V. vinifera* species average when expressed as mg/kg of grapes or mg/berry. However, this appears mainly due to the thick Georgian berry skins, and, thus, it is not due to a higher accumulation in this tissue, but to the higher quantity of pigmented tissue itself. In fact, when considering the anthocyanin accumulation in skins, the average Georgian record is 2.77 mg/g of skin, while the species average is nearly twice higher (4.7 mg/g of skin).

## Resistance to Grapevine Fungal Diseases

The grapevine varieties cultivated worldwide belong to the Eurasian grapevine, *V. vinifera*, and are susceptible, at different levels, to several pathogens (fungi, bacteria, and viruses), while non-*vinifera* species, from North American and Asian, are resistant to fungi and tolerant to viruses and some bacteria (Oliver and Fuchs, 2011; Armijo et al., 2016). Amongst the various diseases which directly affect grapevines, powdery mildew (caused by the ascomycete *Erysiphe necator*) and downy mildew (caused by the oomycete *Plasmopara viticola*) are two of the most important (Bois et al., 2017). Disease management became an unavoidable task for European viticulture in the second half of the nineteenth century, when the two pathogens were introduced into Europe and the European grapevine growers were faced with their destructive effects (Töpfer et al., 2011). The *P. viticola* introduction was a probable consequence of the massive importation of American grapevine species to be used as rootstock for *V. vinifera* and contrast the destructive effects of phylloxera, caused by *Daktulosphaira vitifoliae*, on the Eurasian grapevine species (Granett et al., 2001; Gessler et al., 2011). The search for suitable tools for disease management rapidly became a priority for the viticulturists. The discovery of the efficacy of sulfur and copper in controlling the diseases was a key point, but great attention was also paid to the development of resistant cultivars. The American *Vitaceae* soon proved to be the best sources of resistance, due to co-evolution with the pathogens, and extensive breeding programs, based on interspecific crosses between American *Vitis* species (e.g., *Vitis riparia*, *Vitis rupestris*, *Vitis berlandieri* and *Vitis labrusca*) and *V. vinifera*, were undertaken at the beginning of the XX century (Gessler et al., 2011). Nevertheless, the interest in searching for resistant plants decreased over time, probably due to the discovery of new fungicides (Russell, 2005), that were widely employed for disease control, and the inheritance of the specific foxy off-flavors from the non-*vinifera* parent species.

Recently, public concern about sustainability in agriculture and newregulations on plant protection products have renewed the interest ofgrowers in the cultivation of resistant varieties (Merdinoglu et al., 2018). In fact, although viticulture in the whole of the EU only occupies alow percentage of arable land, the industry is responsible for a highuse of fungicides to fight downy mildew infections (Eurostat^2^). Furthermore, studies on the effects of CO_2_ and temperature on downy and powdery mildews showed that the disease incidence of downy mildew increases with rises in gas and temperature, while an increase in CO_2_ did not influence powdery mildew incidence (Pugliese et al., 2011). In view of the coming climate change, that will potentially favor the pathogens’ development, it is also important to search for new resistance genes, focusing on alternative species, such as *V. vinifera*, to the non-*vinifera* ones.

### *V. vinifera* Resistant Cultivars Against *P. viticola*

The identification of *P. viticola* dates back to 1838, when Schweinitz, one of the founders of American mycology, collected the first samples from wild *Vitis* species in South Carolina (Gessler et al., 2011). In Europe, downy mildew was first reported during 1878 in Bordeaux and then it spread all over the old continent and beyond, reaching Australia and New Zealand between 1919 and 1926 (Emmett et al., 1992). All traditional European grapevine cultivars showed high susceptibility to the pathogen, leading to severe pandemics across Europe (Boso and Kassemeyer, 2008; Gessler et al., 2011). Today, the pathogen is found in warm and humid climates worldwide.

**FIGURE 5:**
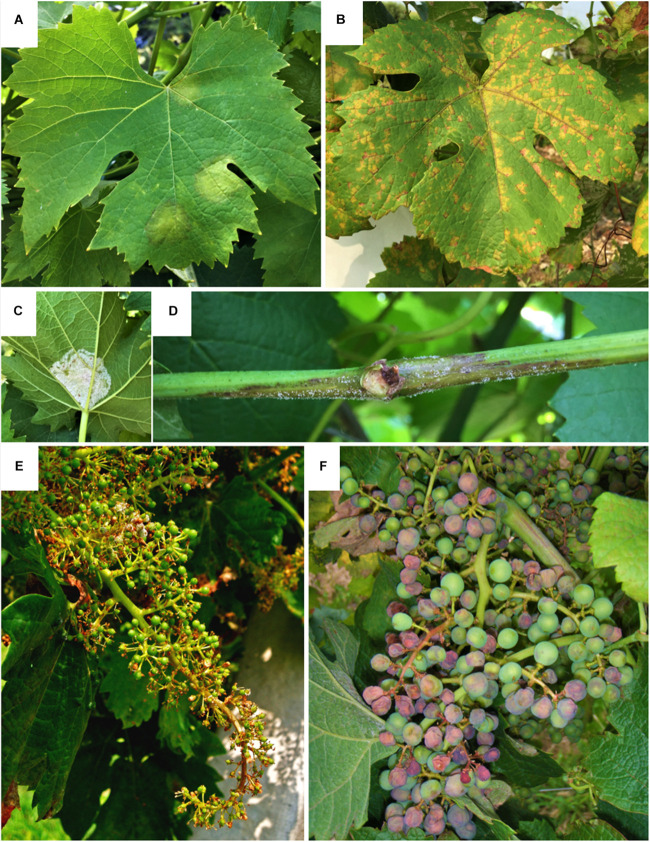
Symptoms of grapevine downy mildew on leaves **(A–C)**, shoot **(D)** and bunches **(E,F)**. **(A)** Oilspot (yellow circular spots with an oily appearance) on the upper side of the leaf; **(B)** mosaic symptom (yellow spot restricted by veins to form yellow-to-brown small, angular spots in a mosaic pattern) on the upper side of the leaf; **(C)** sporulation (sporangiophores and sporangia appearing as a bright white, fluffy growth) on the undersides of leaves; **(D)** shoot covered by sporulation turning brown; **(E)** distorted bunch (U-shaped) turning necrotic; **(F)** shrinking berries turning violet.

Symptoms of downy mildew (Figure 5) are observable on infected organs as yellowish oily lesions (sometimes red, in black cultivars) on the upper surface of the leaves (Figures 5A,B) followed by sporulation on the underside of the leaf (Figure 5C); malformations and necrosis on herbaceous shoots and inflorescences (Figures 5D,E); change of color to violet and withering on berries (Figure 5F), that detach from the rachis leaving a dry stem scar (Gessler et al., 2011). The disease negatively impacts grape production at both qualitative and quantitative levels: the loss of photosynthetic tissues limits the sugar amount in berries, that produce low quality wines; the shoot and bunch damage leads to poor yields. Severe infections, in the absence of disease control, can result in total loss of leaves and in some cases, total yield loss (Töpfer et al., 2011; Toffolatti et al., 2018).

Most of the *Vitis* taxa native to North America are to some extent resistant to *P. viticola* (Unger et al., 2007). The resistance response to *P. viticola* results in rapid plant cell death after pathogen recognition and local necrosis induction. This mechanism, known as the hypersensitive response (HR), is an actively triggered procedure initiated by fungal elicitors or other elicitors (Balint-Kurti, 2019) that leads to bursts of production of ROS and nitric oxide (NO). Consequently, the host cells collapse and shrink, hampering the fungal infection (Toffolatti et al., 2016). Cell death is visible to the naked eye as small necrotic spots on plant tissues.

The Georgian grapevine germplasm is characterized by very high genetic diversity, with cultivars differing from major European ones (Imazio et al., 2013). Considering that this high variability could also be a source of resistance to important pathogens, studies have been undertaken to assess the resistance levels of Georgian accessions to *P. viticola*. The first one, carried out by Bitsadze et al. (2015), showed that 20 accessions were characterized by medium to high levels of resistance to downy mildew in a collection of 61 native Georgian varieties. Given the promising results, it appeared worthwhile to keep screening Georgian germplasm. In Toffolatti et al. (2016), a total of 93 accessions were studied over a period of 3 years in field surveys and in the laboratory. A small group of varieties, including Kamuri Shavi, Mgaloblishvili and Ubakluri, showed low disease severity values, but only Mgaloblishvili showed a strong and constant phenotypical resistance against the pathogen. In Supplementary Table 1, a list of Georgian resistant varieties is reported. Indeed, recent studies on the transcriptome of Mgaloblishvili showed that the cultivar possesses a unique response to *P. viticola* that is based on the overexpression of genes that are not modulated or downregulated in susceptible (Pinot Noir, a *V. vinifera* cv) and resistant (Bianca, interspecific hybrid) cultivars (Toffolatti et al., 2018). The resistance mechanism of Mgaloblishvili is based on the overexpression of genes encoding: (i) receptors for pathogen recognition (PAMP – Pathogen Associated Microbial Patterns-receptors) and for damage at the cell wall (DAMP – Damage Associated Microbial Patterns); (ii) an NB–LRR receptor of fungal effectors (named Lr10); (iii) ethylene signaling; (iv) synthesis of terpenes, such as valencene, and flavonoids; and (v) strengthening of cell walls. Besides genes involved in resistance, susceptibility genes were identified as well. Susceptibility genes are essential for plant-pathogen interaction and their disruption leads to resistance, as with *mlo* gene, whose knockdown is involved in resistance to *E. necator* (Pessina et al., 2016). The candidate gene related to susceptibility to *P. viticola* in *V. vinifera* encodes an LOB domain-containing (LBD) protein (Toffolatti et al., 2020) that has been previously found in the interaction between *Arabidopsis thaliana* and *Fusarium oxysporum* (Thatcher et al., 2012). The new genome editing tools, providing several protocols to introduce knockout on target sequences, makes the understanding of plant pathogen-resistance mechanism mediated by susceptibility genes a very attractive alternative for the development of durable disease-resistant varieties (Zaidi et al., 2018).

#### New Resistant Loci Associated With Resistance to *P. viticola* in *V. vinifera*

**FIGURE 6:**
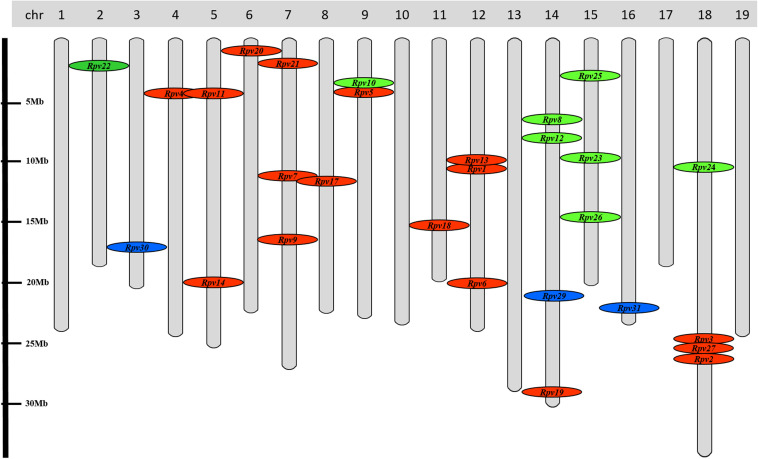
Distribution of resistance loci to *P. viticola* (*Rpv*) overall the 19 grapevine chromosomes. In red: loci identified in Northern American *Vitis* species. In green: loci identified in Asian *Vitis* species. In blue: loci identified in *V. vinifera* genetic background (Georgian germplasm). No information is now available for *Rpv15* and *Rpv16* detected in *Vitis piasezkii* Maxim (Pap et al., unpublished).

The investigation of the genetic basis of *P. viticola* resistance through QTL (Quantitative Trait Loci) analysis on a range of North American and Asian *Vitis* species has led to the identification of 28 resistance (R) loci (Figure 6). These R loci (designated Rpv for Resistance to *P. viticola*) confer different degrees of resistance to disease, ranging from partial to total resistance (Dry et al., 2019). The major loci on this list are: (i) Rpv1, identified in *Muscadinia rotundifolia*, that confers a not total resistance to *P. viticola* infection and is associated with a gene encoding a TIR-NB-LRR protein (MrRPV1) (Merdinoglu et al., 2003; Feechan et al., 2013); (ii) Rpv2, identified in *M. rotundifolia*, that confers total resistance to downy mildew and is associated to a cluster of TIR-NB-LRR genes (Dry et al., 2019); (iii) Rpv3, identified in *V. labrusca, Vitis lincecumii*, *V. riparia* and *V. rupestris*, that confers partial resistance to downy mildew (Bellin et al., 2009; Gaspero et al., 2011; Welter et al., 2017); (iv) Rpv8 and Rpv12, identified in *V. amurensis*, that confer a high resistance to *P. viticola* infection and are associated with the cluster of genes encoding NB-LRR proteins (Blasi et al., 2011; Venuti et al., 2013); (v) Rpv15, identified in *Vitis piasezkii*, that confers strong resistance to *P. viticola* infection (Dry et al., 2019). The other R loci are considered minor loci due to their ability to confer low degrees of resistance and they are only useful when combined with major R loci.

Very recently, new promising downy mildew R loci (Rpv29, Rpv30, and Rpv31) have been identified, through a GWAS (Genome Wide Association Study), in the genetic background of the Georgian *V. vinifera* germplasm (Figure 6) (Sargolzaei et al., 2020). These new R loci, mapping on chromosome 14, 3 and 16 for Rpv29, 30 and 31, respectively, and conferring from high to very high resistance to downy mildew, seem to be associated with receptors of pathogen effectors, signaling mediated by protein ubiquitination and a cluster of Lr10-like (NB-LRR) effector receptors.

### Low Susceptibility of Georgian Grapevine Cultivars to Phytoplasma-Associated Diseases

Flavescence dorée (FD) and Bois noir (BN) are the more important diseases of the grapevine yellows (GY) complex, responsible for severe yield losses in vineyards worldwide (Belli et al., 2010). FD and BN are associated with phytoplasmas, phloem-limited bacteria transmitted by insect vectors (Weintraub and Beanland, 2006). Even if their symptoms were indistinguishable (desiccation of inflorescences, berry shrivel, leaf discolorations, reduction of growth, and irregular ripening of wood), FD and BN are associated with phytoplasmas distinct at both genetic and ecological level (Belli et al., 2010). The FD phytoplasma is efficiently transmitted from grapevine to grapevine by the insect *Scaphoideus titanus*, which sustains its whole life cycle on *Vitis* spp. (Oliveira et al., 2019). Consequently, geographic areas hosting large vector populations and FD phytoplasma can be damaged by strong FD epidemics. Due to this aspect, FD phytoplasma is a quarantine pathogen, to be controlled through mandatory measures (Oliveira et al., 2019). On the other hand, BN phytoplasma (‘*Candidatus* Phytoplasma solani’) (Quaglino et al., 2013) is occasionally transmitted to grapevine by the insect *Hyalesthes obsoletus*, a polyphagous vector living preferentially on *Urtica dioica* (nettle), *Convolvulus arvensis* (bindweed), and *Vitex agnus-castus* (chaste tree) (Langer and Maixner, 2004; Kosovac et al., 2016). The epidemiological cycle associated with BN is extremely complex and was recently discovered to include other highly polyphagous insect vectors and a very broad range of secondary wild hosts (Mori et al., 2015; Quaglino et al., 2019). Moreover, the typical management strategies for phytoplasma diseases, based on the control of the vector(s) with insecticides and the removal of infected plants, are not effective against BN. Thus, it is difficult to organize effective prevention and containment measures. An ambitious strategy is based on the selection of plant varieties as the source of resistance-genes for plant breeding programs (Bianco et al., 2019). Unfortunately, none of the *Vitis* species and *V. vinifera* varieties studied have been found to be resistant or tolerant to the GY phytoplasmas (Laimer et al., 2009).

**FIGURE 7:**
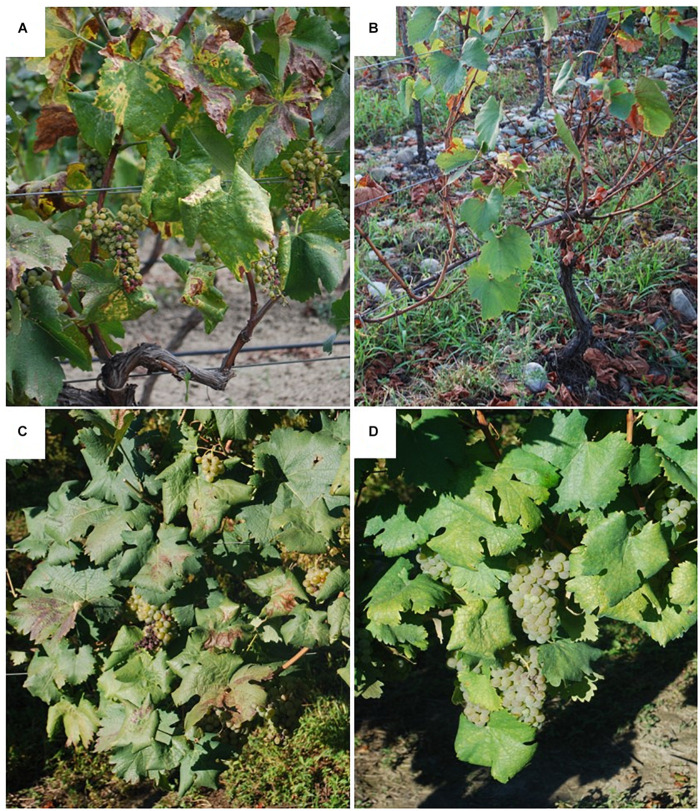
Symptoms observed on ‘*Candidatus* Phytoplasma solani’ infected grapevine cultivars in Georgia. Severe symptoms on international cultivar Chardonnay **(A)** and Georgian cultivar Kisi **(B)**; moderate symptoms on Georgian cultivar Goruli Mtsvane **(C)**; mild symptoms on Georgian cultivar Tsitska **(D)**.

Surveys conducted in vineyards of Khaketi and Shida Kartli regions in eastern Georgia highlighted a wide diffusion of BN, while FD was not reported (Quaglino et al., 2014). Moreover, most autochthonous Georgian grapevine cultivars were found to be only mildly symptomatic, maintaining complete berry production, while internationally known cultivars exhibited severe symptoms (Quaglino et al., 2016) (Figure 7). As largely reported for phytoplasma-associated diseases of stone fruits, symptom intensity observed in infected plants can be influenced both by the virulence of the pathogen and the susceptibility level of the plant host (Kison and Seemuller, 2001; Seemüller and Schneider, 2007). Molecular characterization, supported by phylogenetic analyses, revealed that BN phytoplasma strains identified in Georgia constitute a bindweed-related population which is genetically distinct from the one found in central-western Europe. Interestingly, the presence of the same phytoplasma strain in grapevine cultivars showing a range of symptom intensity suggested a low susceptibility of Georgian local cultivars to BN (Quaglino et al., 2016) (Supplementary Table 1). Studies in progress are focusing on (i) identifying genetic traits associated with this low susceptibility to BN in the perspective of improving breeding programs to produce novel tolerant and/or resistant grapevine cultivars; (ii) investigating the susceptibility of Georgian grapevine cultivars to FD.

## A Brief Interlude on the Status of Georgian Wild Compartment

The *V. vinifera* subsp. *silvestris* is considered the progenitor of cultivated species. In the last two decades, an increase interest in preserving wild genetic resources has led to surveys on Georgian land aimed to localize and gather wild grapevine material. The plant material collected in these surveys is summarized in the Supplementary Table 2. These accessions are now partially (more than 100) available in Georgian collections (Saguramo, Skra and other collections) and some other in USDA National Clonal Germplasm Repository of Davis (CA, United States) and in the collection of Milan University. This subspecies is seriously worldwide endangered by human activities, such as urbanization, forest cleaning and setting fires (Arnold et al., 2005). The Georgian one is not an exception. Indeed, very small wild populations have been identified overall the Georgian land (Ocete Rubio et al., 2012). Populations with both male and female individuals were detected, but in Zhinvali and Sabue populations no female individuals were identified. Generally, in the Georgian populations the number of male individuals is higher than the female ones (Supplementary Table 2). Most of the wild Georgian populations showed severe downy and powdery mildew symptoms, although three individuals showed high resistance to *P. viticola* infection (Supplementary Table 2) (Ocete Rubio et al., 2012; Bitsadze et al., 2015). Nevertheless, remarkable is the absence of symptoms caused by phylloxera in the populations sampled by Ocete Rubio et al. (2012). In the same populations, symptoms caused by two mites, *Colomerus vitis* and *Calepitrimerus vitis*, have been observed, although the damages were not serious and appeared to do not affect the viability of the plants.

From the genetic point of view, some of these accessions weregenotyped by SSR and SNP molecular markers(Supplementary Table 2) (Imazio et al., 2013; Ekhvaia et al., 2014; De Lorenzis et al., 2015; Riaz et al., 2018). Results reported in Imazio et al. (2013) and De Lorenzis et al. (2015) clearly discriminatedthe wild individuals from the cultivated ones, two subspecies that diverged at least 22,000 years ago (Zhou et al., 2017; Liang et al., 2019). The Georgian accessions were differentiated by European wild accessions and cultivated accessions (Riaz et al., 2018). Interesting, Ekhvaia et al. (2014) identified absence of genetic isolation among some of the analyzed wild populations due to gene flow among them.

At the phenotypical level, few information is available and further studies need to deeply investigate the enological potential of this compartment. Nevertheless, preliminary results showed that musts obtained by Georgian wild grapes could be added to the must of traditional cultivars to improve the wine color (Maghradze et al., 2020).

## What’s Next?

Climate change will impact many aspects of human life, the environment, agriculture and food. Regarding viticulture, data available on climate change have already demonstrated impacts on wine growing areas, resulting in changes in grape chemical composition as well as grape phenology. Because of their isolated geographical origin and huge genetic variability, the Georgian grapevine germplasm is of great interest as a worthwhile resource for breeding programs. The Georgian germplasm has distinguished itself by including cultivars characterized by late ripening, which could potentially reduce issues related to excessive temperatures in summertime, distinctive eno-carpological traits, which affect the grape and wine quality, specific response to abiotic stresses, such as sunburn, and resistance traits related to biotic stresses, such as *P. viticola* and phytoplasmas.

Given the reasons stated in this review, the screening and assessment of Georgian germplasm should be promoted at the phenotypical, agronomical, physiological and genetic level. A number of gaps has still to be filled, such as their attitude to abiotic (drought, salinity, iron chlorosis) and biotic stresses, as well as the whole genome analysis of the most performing Georgian cultivars, in order to identify the genetic regions related to such valuable traits. A step toward this direction has been performed by Tabidze et al. (2017), sequencing the whole genome of four major Georgian varieties (Chkhaveri, Saperavi, Meskhetian green, and Rkatsiteli) and releasing information useful to understand the complexity of grape genome and for further comparative analysis. Aside from traditional breeding programs, these invaluable resources could be exploited in breeding programs based on the use of New Breeding Technologies (NBTs), by means of genome editing applied to both resistance and, with even more practical advantages, susceptibility candidate genes to abiotic and biotic stresses. In this way, it will be possible to exploit the valuable traits carried by this unique source of genetic variability for new varieties able to meet the challenges awaiting viticulture in the era of climate change.

## Author Contributions

GDL and SLT conceived the work. MS, VR, and GDLwrote the introduction and genetic variability section. GC wrote theclimate section. LR wrote the section on phenotype. SLT wrote the section on resistance to fungal pathogens. FQ wrote the section on susceptibility to phytoplasma diseases. DM, OF, and PAB critically revised the manuscript. All the authors read and approved the final version of the manuscript.

## Conflict of Interest

The authors declare that the research was conducted in the absence of any commercial or financial relationships that could be construed as a potential conflict of interest.

## Footnotes

1 http://www.oiv.int/

2 http://ec.europa.eu/eurostat/de

## Supplementary Material

Supplementary Table 1 List of Georgianautochthonous and breeding grapevine accessions based on Ketskhoveliet al. (1960) and VIVC. Table includes information related to berry color, origin, usage, spreading, genotyping and resistance to plant disease. Synonyms, homonyms and misidentifications are not verified. SSR, simple sequence repeat; SNP, single nucleotide polymorphism; VIVC, Vitis International Variety Catalogue (https://www.vivc.de/).

Supplementary Table 2 List of Georgian wild grapevine accessions. Table includes information related to flower sex, origin, genotyping and resistance to plant disease. SSR, simple sequence repeat; SNP, single nucleotide polymorphism; VIVC, Vitis International Variety Catalogue (https://www.vivc.de/). F, female; H, hermaphrodite; M, male; UNIMI, University of Milan.
